# Neural Markers of Attention at 6 Months Associate With Later Attentional Control Performance

**DOI:** 10.1111/desc.13582

**Published:** 2024-11-06

**Authors:** Alexandra Hendry, Manuela Stets, Pasco Fearon, Mark Johnson, Karla Holmboe

**Affiliations:** ^1^ Department of Experimental Psychology University of Oxford Oxford UK; ^2^ Department of Psychology University of Essex Colchester UK; ^3^ Centre for Family Research University of Cambridge Cambridge UK; ^4^ Department of Psychology University of Cambridge Cambridge UK; ^5^ School of Psychological Science University of Bristol Bristol UK

**Keywords:** attention, EEG, ERP, executive function, infant

## Abstract

Attentional control is key to the development of executive functions. Previous research indicates that individual differences in attentional control behaviour may be stable from 6 months. Here, we analyse electroencephalogram data collected from 59 6‐month‐olds to gain insights into the neural processes underlying attentional control in infancy. First, we examine the neural activity preceding distinct looking behaviours in an attentional control task at 6 months. Second, we test whether those neural markers show predictive associations to behavioural measures of attentional control (Freeze‐Frame task) and executive function (A‐not‐B task) in the same infants at 9 months. Whilst our data do not show evidence that 6–9 Hz power is implicated in attentional control at 6 months, or that the P1 ERP component plays a role in our attentional control task, we do find evidence that corroborates and extends research linking 3–6 Hz power to attentional control. At the group level, frontal 3–6 Hz power recorded whilst looking to a central target before the onset of a peripheral distractor was greater during trials where infants subsequently looked to the distractor, compared with trials where they did not look. Higher 3–6 Hz power in trials where the infant *did not* look to a peripheral distractor was predictive of less distractibility at 9 months, and higher 3 Hz power in trials where infants *did* look to the distractor strengthened the predictive association from 6‐month EEG to 9‐month behaviour. We suggest 3–6 Hz activity may be sensitive to multiple processes, such as anticipatory attention, and the ability to maintain attention on a target.

## 1 Introduction

### 1.1 Early Development of Attentional Control

Attentional control involves the maintenance or (de)prioritisation of one or more stimuli or cognitive tasks for active processing. Attentional control is thus a core mechanism by which infants manage the multitude of competing inputs which might otherwise overwhelm their cognitive capacities. In particular, the ability to maintain attentional focus and to resist distraction enables infants to maintain engagement with a target for the purpose of active information processing (Hendry, Johnson, and Holmboe 2019). This ability develops within the first year, with infants aged 6 months and older being less distractible when in a state of focused attention (as defined by behavioural indicators of active object examination or heart‐rate indicators of sustained lowered heart rate) compared with when they are in a state of casual attention (Lansink and Richards 1997; Oakes, Ross‐Sheehy, and Kannass 2004; Richards and Turner 2001; Ruff, Capozzoli, and Saltarelli 1996). The duration of individual epochs of focused attention during free play increases during the first year, and appears to be increasingly driven by endogenous cortical processes (Amadó et al. 2023).

**Summary**

- In 6‐month‐old infants, frontal EEG 3–6 Hz power was greater during trials where infants subsequently looked to a distractor (compared with trials where they did not).
- Higher 3–6 Hz power in trials where infants *did not* look to a peripheral distractor was predictive of less distractibility at 9 months.
- Higher 3 Hz power in trials where infants *did* look to a distractor strengthened the predictive association from 6‐month EEG to 9‐month behaviour.
- 3–6 Hz EEG power may be sensitive to multiple processes in infancy, including anticipatory attention and the ability to maintain attention on a target.

By 31 months, weak focused attention during free play is associated with greater distractibility on another task, with more limited evidence for such inter‐task associations at 9 months (Kannass, Oakes, and Shaddy 2006). The limited inter‐ and intra‐task associations in looking behaviour during infancy could be taken as indicating that attentional control is not a unified or stable construct at this age. Alternatively, it may be the case that, in addition to attentional control, looking behaviour in contexts such as object exploration is influenced by a broad range of factors in the first years of life (e.g., processing speed, motivational factors); the impacts of which are likely to vary from task to task (Hendry, Johnson, and Holmboe 2019).

Indeed, within the context of more‐constrained looking behaviour tasks, such as eye‐tracking tasks, there is evidence for continuity in infancy and toddlerhood. Among 2‐year‐olds, performance on an antisaccade eyetracking task (in which participants are rewarded to suppress automatic saccades to a distractor and instead execute an anticipatory saccade in the opposite direction) is significantly correlated with performance on a reversal learning eyetracking task (which captures toddlers’ ability to control their anticipatory saccades according to a new rule, following a switch from a previous opposing rule; Hendry et al. 2024). Even earlier than this, 6‐month‐olds’ looking behaviour on the Freeze‐Frame eyetracking task—in which peripheral distractors are presented intermittently alongside a more visually complex and changing central stimuli—is predictive of Freeze‐Frame looking behaviour at 9 months (Holmboe et al. 2018).

### 1.2 Executive Attention as a Common Mechanism in Attentional Control and Executive Functions

Executive functions are the higher‐order and top‐down cognitive skills that enable us to control behaviour in pursuit of a goal. These skills include impulse control, inhibition of a prepotent response, updating or manipulating content held in short‐term memory and response shifting. In infancy and toddlerhood, executive functions can be measured through parent report of goal‐directed behaviour (Hendry and Holmboe 2021) or through performance on tasks that require some combination of the skills listed above, such as the A‐not‐B task further detailed below (Hendry, Jones, and Charman 2016; Morgan, Fiske, and Holmboe 2025).

Individual differences in executive functions, and in the ability to maintain endogenous attentional control, have been linked to a common limited‐capacity cognitive mechanism, sometimes referred to as executive attention. This mechanism is implicated in the goal‐ directed control of both attention and behaviour in contexts of novelty and interference (Hendry, Johnson, and Holmboe 2019; Rueda, Posner, and Rothbart 2005; Tiego et al. 2020). In support of this idea, looking behaviour on the Freeze‐Frame task at age 9 months is associated with concurrent performance on the A‐not‐B task (Holmboe et al. 2018).

Indices of endogenous attentional control in infancy have been demonstrated to show some longitudinal associations from age 10 months to behavioural measures of executive function in toddlerhood, which may be seen as further evidence for the role of executive attention in endogenous attentional control (Frick et al. 2018; Johansson et al. 2016; Johansson et al. 2015). Yet, only a weak, non‐significant, association is observed between 6‐month Freeze‐Frame and 9‐month A‐not‐B task performance (Holmboe et al. 2018). It is plausible that executive attention only develops as a common mechanism linking attentional control to executive functions from around 9 months. However, given the predictive associations described above between looking behaviour within the Freeze‐Frame task at 6 and 9 months, and between Freeze‐Frame and A‐not‐B performance at 9‐months, it is also plausible that executive attention is present as an underlying mechanism whose common influence longitudinally across attentional control and executive function tasks is masked by measurement noise (i.e., due to different, non‐attentional influences on looking behaviour on Freeze‐Frame versus reaching behaviour on A‐not‐B). Honing in on possible neural indices of attentional control may help resolve this ambiguity.

### 1.3 Neural Indices of Attentional Control in Infancy

Research into the neural basis of infant attentional control has primarily relied upon electroencephalogram (EEG) data. The EEG signal can be analysed in two different ways: event‐related potentials (ERPs) and EEG power (Bullock 1989). We focus primarily on EEG power, but also had questions relating to ERPs (specifically the P1 component); for transparency, the context and analysis relating to these questions are presented in Supplementary Materials 1 (SM1). To measure EEG power, researchers first decompose the EEG signal into separate frequency bands, which reflect populations of neurons firing together and being inhibited together in synchrony. Particular rhythms or frequency bands have been suggested to associate with specific types of cognitive function. Research on infant attentional processes has tended to focus on two frequency bands: alpha (circa 6–9 Hz) and theta (circa 3–6 Hz) although there is still some debate over the appropriateness of these labels and cut‐offs, and borderline frequencies (5.6–6.0 Hz) may display properties of both types of activity (Orekhova, Stroganova, and Posikera 2001). For this reason, and to aid with interpreting the somewhat contradictory findings in the literature to date, we refer explicitly to the frequencies used in the analyses reviewed below.

#### 1.3.1 6–9 Hz Activation in Anterior and Posterior Scalp Locations Is Associated With Top‐Down Control During Visual Attention

Although engagement of visual attention is often considered in terms of alpha (6–9 Hz) suppression (see Klimesch 1999, 2012 for review), the paradigms used in the infant studies in which reduction in 6–9 Hz power is observed tend to involve externally evoked visual attention in noncognitively–demanding contexts such as watching an adult blowing bubbles, or a puppet character singing and dancing; that is, not necessarily engaging top‐down control (Stroganova, Orekhova, and Posikera 1999; Xie, Mallin, and Richards 2018). In contrast, infant research indicates that 6–9 Hz *increases* may reflect active top‐down inhibition of task‐irrelevant brain regions in order to achieve internally directed goals (again, consistent with the adult literature [Klimesch 2012; Vissers, van Driel, and Slagter 2016]). Amongst 8‐ to 11‐month‐olds, anticipatory, internally controlled attention is associated with relatively *higher* 6.8 Hz power at posterior parietal sites (Orekhova, Stroganova, and Posikera 2001). During completion of an A‐not‐B task (which, as noted above, can be considered to have executive attention demands), 8‐month‐olds show an increase in 6–9 Hz power compared to EEG collected during passive visual attention (Bell 2002; Bell and Wolfe 2007) and 6–9 Hz power discriminates infants' correct and incorrect responses during the task (Bell 2002). These effects are observed at both anterior and posterior scalp locations (Bell 2002). There is however a dearth of research into the longitudinal correlates of changes in 6–9 Hz power in response to the demands of a task that elicits top‐down attentional control amongst infants younger than 8 months.

#### 1.3.2 3–6 Hz Activation in Anterior Scalp Locations Is Associated With Top‐Down Control During Visual Attention

The second frequency band commonly associated with attentional control is theta. In adults and children (> 4 years), it has been suggested that theta activity in the frontal brain areas may function in attention maintenance, monitoring and control (Cavanagh and Frank 2014; Clayton, Yeung, and Cohen Kadosh 2015; Senoussi et al. 2022; van Noordt, Heffer, and Willoughby 2022). For example, increases in fronto‐medial theta have been associated with presentation of rare oddball stimuli, and with post‐error enhancements in performance amongst adults (Clayton, Yeung, and Cohen Kadosh 2015), and with interference control and response inhibition demands amongst children (Adam et al. 2020). In the infant literature, frontal theta power—most commonly operationalised as 3–6 Hz EEG—has also been linked to deployment of top‐down attentional control and increased information processing (Begus and Bonawitz 2020; Saby and Marshall 2012). For example, task‐related increases in frontal 3–6 Hz power have been observed during eye gaze processing, expectation of incoming information, prediction error encoding, and the progressive encoding and depletion of information processing (Begus, Southgate, and Gliga 2015; Köster, Langeloh, and Hoehl 2019; Köster et al. 2021; Michel et al. 2015; Piccardi, Johnson, and Gliga 2020). Although varied, these paradigms may have in common the engagement of top‐down control to guide or gate other bottom‐up cognitive processes in service of learning and other goal‐directed behaviour. Where more‐generalised or posterior theta increases are found, these may be attributable to emotional reactivity (Saby and Marshall 2012; Stroganova and Orekhova 2013).

With regards to attentional control specifically, heart‐rate defined sustained visual attention is associated with increased power in the 4–6 Hz range in frontal regions amongst 3‐month‐olds (Brandes‐Aitken et al. 2023), and in the 2–6 Hz range in frontal, temporal and parietal regions from 10 months onwards (Xie, Mallin, and Richards 2018). At 10 months, relative 3–6 Hz power across frontal, central and occipital regions 0–2000 ms after onset of a new attention episode during object play predicts the subsequent duration of that attention episode (Amadó et al. 2023). Frontal 3–7 Hz power during 12‐month‐olds’ independent object play has been shown to predict the duration of visual fixation such that higher theta power in the 1000 ms before look onset is positively associated with the subsequent duration of that look (Wass et al. 2018). Amongst 8‐ to 12‐month‐olds (6–12 months corrected for gestational age), researchers observed increases in power (from a baseline of quiet attention to bubbles) in the 3.6–5.6 Hz range, in response to self‐generated focused attention during object exploration and in response to attention during infant‐directed speech. Exploratory follow‐up indicated that increases in 4.0–4.4 Hz oscillations (identified as the peak theta frequency) were localised to frontal regions, whereas increases at the high boundary of the range were localised to posterior temporal regions during manipulation, and temporo‐parieto‐occipital regions during speech and may be indexing some function other than attentional control (Orekhova et al. 2006).

#### 1.3.3 3–6 Hz Activation Patterns Vary Across the Duration of a Task, Potentially Indexing Information Processing

Just as attention to stimuli may change with exposure (Stets and Reid 2011), theta may vary over the course of a task. For example, over the course of viewing a 1‐ to 3‐min video, 3–6 Hz power increases in frontal regions have been observed amongst 6‐, 10‐ and 12‐month‐olds (Braithwaite et al. 2020; Jones et al. 2020; Piccardi, Johnson, and Gliga 2020), and amongst 9‐month‐olds over the course of 1.3 min of watching bubbles being blown (Stroganova and Orekhova 2013). One interpretation of these results, based on the premise that 3–6 Hz power indexes top‐down attentional control, is that, as stimuli become more familiar and less exogenously captivating over time, infants increasingly engage endogenous control (i.e., voluntary, presumably top‐down control) to maintain their attention on the target. However, if such endogenous control were attributable to an executive attention system, we would expect a significant association between 3 and 6 Hz increases throughout a stimulus presentation at 6 months and measures of executive function and attentional control at 9 months; an association that was tested and not found in the Braithwaite et al. (2020) study. Further, in the Piccardi, Johnson, and Gliga (2020) study, changes in 4–6 Hz power were non‐linear, showing an initial increase from 100 to 200 s compared with 0–100 s, followed by a *decrease* from 200 to 300 s, with no further significant changes in the final period (300–400 s). The authors speculate that when presented with new information (i.e., the unfamiliar video clip), infants may have initially explored the scene, before fully engaging with its contents to extract information about particular aspects of the video (leading to the increase in theta), after which point, presumably, engagement and information processing wanes. This interpretation of 3–6 Hz power as indexing information processing, after an initial period of familiarisation, is compatible with the finding in both the Braithwaite et al. (2020) and Jones et al. (2020) study that individual differences in increases of 3–6 Hz power whilst viewing relatively complex, dynamic stimuli were positively correlated with non‐verbal cognitive ability. However, it seems likely that 3–6 Hz power's utility as a marker of information processing will interact with the nature of the stimuli used, as well as the duration of the presentation.

#### 1.3.4 Longitudinal Associations From 3–6 Hz Activation Differ With Age

To further complicate matters, some studies have found that the direction of effects changes with age in the 3–6 Hz power range. This may be linked to changes in the demands of a task as infants mature. For example, at 7–8 months, frontal 4–5 Hz power is positively correlated with duration of anticipatory attention, with activity in this frequency range significantly greater over frontal regions during anticipatory attention compared to periods of quiet attention (Orekhova, Stroganova, and Posikera 1999; Stroganova, Orekhova, and Posikera 1998). By 9–10 months, however, 3.6–4.8 Hz power is negatively correlated with duration of anticipatory attention—a finding that the authors attribute to a reduction in the amount of frontal activation needed to maintain anticipatory attention at 9 months (Orekhova, Stroganova, and Posikera 1999). An alternative explanation is that for younger infants there is still some prediction error involved in peek‐a‐boo—that is, infants are surprised that the face reappears, and theta increases are a marker of the need to maintain anticipatory attention to learn more about the unfolding events—as has been observed for 4–5 Hz power increases amongst 9‐month‐olds using a violation of expectation task (Köster, Langeloh, and Hoehl 2019; Köster et al. 2021)—whereas by 9–10 months infants tend to have sufficient object permanence that the face reappearing is the expected outcome. Similarly, changes in 4 Hz power at fronto‐central electrodes are associated with changes in gaze towards or away from an object at 5 months but not at 2 or 4 months (assumed to be before the development of executive attention networks) or at 9 months (by which point modulation of attention to gaze shifts may be trivially easy for infants, or already a well‐learned response) (Michel et al. 2015).

### 1.4 The Current Study

The findings summarised above highlight that 6–9 Hz and 3–6 Hz power (the latter particularly in frontal regions), show promise as markers of cognitive function in infancy—but that further research is needed to verify how to interpret these markers. One difficulty in interpreting infant EEG is that studies have to be conducted without verbal instructions to the participants. Therefore, as indicated above, interpretations of changes in power as related to the engagement of top‐down control in service of goal‐directed behaviour must be somewhat speculative, particularly during passive‐looking tasks (Meyer et al. 2019). By considering EEG data collected during a task structured to enable differentiation between different looking behaviours relating to attentional control, alongside longitudinal behavioural measures of attentional control and executive function, we aim to add clarity to the interpretation of infant EEG about early attentional control development.

In the current study, we use the Freeze‐Frame task for collecting behavioural (at 6 and 9 months) and neural (at 6 months only) indices of attentional control, and the A‐not‐B task as a behavioural measure of executive function at 9 months. Neural data collected during the Freeze‐Frame task is considered in terms of two different *looking conditions*: looking to a peripheral distractor (as a potential marker of distractibility or of anticipation/engagement); and not looking to a peripheral distractor (as a potential marker of inhibitory control or of low engagement). We aim to use the results of tests of predictive associations from neural activity during the Freeze‐Frame task at 6 months to attentional control and executive function performance at 9 months to disambiguate these interpretations.

Specifically, we ask, as follows:

1. Do we see different patterns in 3–6 or 6–9 Hz power according to looking behaviour during the Freeze‐Frame task?
2. Do we find predictive associations from 3–6 or 6–9 Hz power within looking conditions on the Freeze‐Frame task at 6 months, to behavioural performance on the Freeze‐Frame task at 9 months (above and beyond predictive associations from behavioural performance on the Freeze‐Frame task at 6 months)?
3. Do we find predictive associations from 3–6 or 6–9 Hz power within looking conditions on the Freeze‐Frame task at 6 months to A‐not‐B performance at 9 months?
4. How does the pattern of results identified above help us to interpret the attentional demands of the Freeze‐Frame task?

## 2 Method

### 2.1 Participants

Parents and infants were recruited from a lab database of Greater London families when infants were 4 months old as part of a longitudinal study in which infants were seen at 4, 6 and 9 months (4‐month data not reported here). Most visits occurred within one week of infants reaching their 6‐/9‐month birthday. Ninety‐six infants (49 female) were seen at 6 months old. Of these, 59 (33 female) provided sufficient artefact‐free recordings to compute indices of EEG power (for inclusion in EEG analyses, see ‘EEG processing’ below): only these infants were included in the longitudinal analyses. See Table 1 for sample sizes at each timepoint and for each analysis, and Table 2 for demographic data for the sample.

**TABLE 1:** Sample sizes and attrition rate at each timepoint and for each analysis.

Sample	*N*	Attrition rate
Seen at 6 months old	96	—
Seen at 9 months old	94	2% of the original sample
Contributed valid behavioural data at 6 months	79	18% of the original sample
Contributed sufficient artefact‐free recordings to compute indices of EEG power: Used in analyses of looking condition effects on EEG power	59	39% of the original sample 25% of the sample with valid 6‐month Freeze‐Frame behavioural data
Contributed valid Freeze‐Frame behavioural data at 9 months	86	10% of the original sample
Contributed valid A‐not‐B behavioural data at 9 months	87	9% of the original sample
Contributed valid Freeze‐Frame behavioural data at 9 months and valid EEG data at 6 months: Used in the analysis of neural correlates of attentional control at 6 months as a predictor of attentional control at 9 months	55	43% of the original sample
Contributed valid A‐not‐B data at 9 months: Used in analysis of neural correlates of attentional control at 6 months as a predictor of attentional control and executive function at 9 months	52	46% of the original sample

**TABLE 2:** Demographic data for sample providing sufficient EEG data (*N* = 59).

	Mean	SD	Minimum	Maximum	*N*
Infant age in days at the 6‐month visit	182.12	6.03	174	198	59
Infant age in days at the 9‐month visit	275.14	6.40	266	295	58
Mother's age in years	34.51	5.35	21	47	57
Mother's years of education	18.46	3.45	13	30	55
Father's age in years	35.65	6.11	23	52	54
Father's years of education	17.50	3.31	11	30	51
Ethnicity					
Asian	—				2
Black	—				2
White	—				44
Mixed	—				11

Behavioural data from the 6‐month and 9‐month visits have been reported previously (Holmboe et al. 2018), along with EEG data from a separate task administered before the Freeze‐Frame task at the 6‐month session (Braithwaite et al. 2020). This is the first time the EEG data from the Freeze‐Frame task has been analysed and reported. The original study and this analysis received ethical approval from the Department of Psychological Sciences ethics committee at Birkbeck (ref. no. 2248).

### 2.2 Procedure

#### 2.2.1 Freeze‐Frame Task

Infants, sat on their parent's lap, watched dynamic cartoon animations in the centre of the screen (see Holmboe et al. 2018, for details of apparatus and stimuli). On every trial, after an interval of 1000–2000 ms (jittered to minimise time‐locked anticipatory processes) a white distractor square was presented peripherally on the left or right side of the screen. The distractor subtended 3.2° of visual angle at 13.5° eccentricity from the centre. The duration of the distractor was individually calibrated in 40‐ms steps, starting at 200 ms, until the infant looked to the distractor on two consecutive trials, at which point the distractor duration was fixed. The aim was for infants to complete as many trials as possible at 6 months and at least 80 trials at 9 months, but the session was stopped if an infant became fussy. At 6 months, infants wore an EEG net as described further below.

##### 2.2.1.1 Version for 9‐month‐olds

For 9‐month‐olds, the Freeze‐Frame stimulus presentation involved a mix of interesting (engaging central stimulus) and boring (repetitive central stimulus) trials. For both trial types, the central animation was frozen during distractor presentation and for 600 ms after the distractor. If the infant made a saccade towards the distractor, the experimenter froze the animation for a further 2400 ms to discourage the infant from looking to the distractors.

##### 2.2.1.2 Version for 6‐month‐olds

For 6‐month‐olds, the Freeze‐Frame stimulus presentation involved only interesting trials (engaging central stimulus), to reduce the number of conditions for the EEG analysis. In order to be able to administer trials consistently and rapidly enough to collect a sufficient number of EEG trials, in the version used at 6 months, the central animation froze on all trials during distractor presentation and for 600 ms after distractor presentation (regardless of the infant's looking behaviour), that is, there was no further ‘freeze’ if the infant looked to the distractor.

##### 2.2.1.3 Derived Measures

At both time‐points the proportion of looks to the distractor in interesting trials (only) was used as the performance measure because this allows for inclusion of more data from each participant (Holmboe et al. 2010), and provides a consistent measure across the two age points (Holmboe et al. 2018). Both the performance measure reported in Holmboe et al. (2008, ‘Selective Inhibition’, the difference between looks to the distractors on boring and interesting trials across the first 16 trials) and in Holmboe et al. (2018, the proportion of looks to distractors across all interesting trials) correlate significantly with A‐not‐B performance at 9 months (Holmboe 2008, p. 176).

Video recordings of each infant's looking behaviour were coded offline. A trial was defined as a Look trial if the infant made a saccade toward the peripheral distractor, or as a NoLook trial if the infant maintained gaze on the central stimulus. A trial was considered invalid if the infant was not looking at the central stimulus at distractor onset, blinked during distractor presentation, or if the infant's eyes were out of view for more than two frames (80 ms) during distractor presentation or within 1000 ms following distractor presentation. Trials were also considered invalid if a saccade to the distractor was initiated earlier than three frames (120 ms) after distractor onset (such saccades are considered anticipatory or random). Inter‐coder reliability was excellent for looking behaviour and trial validity (κ = 0.86–0.97), based on all trials from a random sample of 5–10 participants at each age (*N* = 526–750 trials).

#### 2.2.2 Electrophysiological Recording

EEG was recorded in a shielded room using 64‐channel sensor nets from Electrical Geodesics Inc. (EGI), referenced to the vertex and digitized at 250 Hz. Before processing, the EEG data were band‐pass filtered between 0.3 and 30 Hz and re‐referenced to the average of all scalp electrodes, excluding channels previously marked as bad.

##### 2.2.2.1 EEG Processing

Frequency‐bands were analysed for stretches of EEG recorded 800–200 ms before distractor onset (i.e., during attention to the central stimuli). Using EEGLab version 12.0.2.5b (Delorme and Makeig 2004), epochs were visually inspected and rejected if trials had been marked as invalid during video coding. Additional reasons for trial exclusion were contamination by movement or other artefacts. An infant's data were included in the final analysis if they reached a minimum criterion of contributing 8 valid trials to both the Look and the NoLook condition.

The 59 6‐month‐old infants included in the final EEG condition effects analysis contributed an average of 12.86 artefact‐free Look trials (*SD* = 4.33, range 8–25, total 759) and an average of 13 artefact‐free NoLook trials (*SD* = 4.18, range 8–25, total 767). As the majority of Look trials tended to occur in the earlier stages of an infant's test‐session (due to naturally occurring and intended changes in looking behaviour as infants learn about the task, rather than to task design) and in order to keep the trial numbers more equal between conditions, for analysis of condition effects we excluded excess NoLook trials (*M* = 18.08, *SD* = 13.95) for 36 infants. The cut‐off for inclusion of NoLook trials was selected as follows: (i) for a participant contributing 8 or 9 Look trials only, 10 artefact‐free NoLook trials were included in the analyses; (ii) if 10 or more usable Look trials were available, they were matched with the same number of NoLook trials. For eight infants, more useable Look trials had been recorded than NoLook trials and, respectively, excess Look trials (*M* = 7.25, *SD* = 6.5) were excluded from further analyses.

Time‐frequency analyses were performed on each artefact‐free trial by continuous wavelet transform using Morlet wavelets at 1 Hz intervals in the 3–9 Hz range using WTools (developed by E. Parise, L. Filippin, & G. Csibra). Segments were selected from 1600 ms before to 600 ms after the onset of the peripheral distractor to allow for a 400 ms buffer on either end to avoid possible distortions resulting from the wavelet transformation to spill into the time‐window of interest. These 400 ms‐long buffer‐zones were then removed after the transformation leaving epochs starting at −1200 ms before and ending at 200 ms after distractor onset. Activity in the 3–9 Hz frequency range during a baseline period (i.e., −1200 to −1000 ms before distractor onset) was subtracted from activity during the analysis period. We chose this time window as our baseline because at that point the infant would not currently be distracted by the distractor presented on the previous trial and would be attending to the centre of the screen.

**FIGURE 1:**
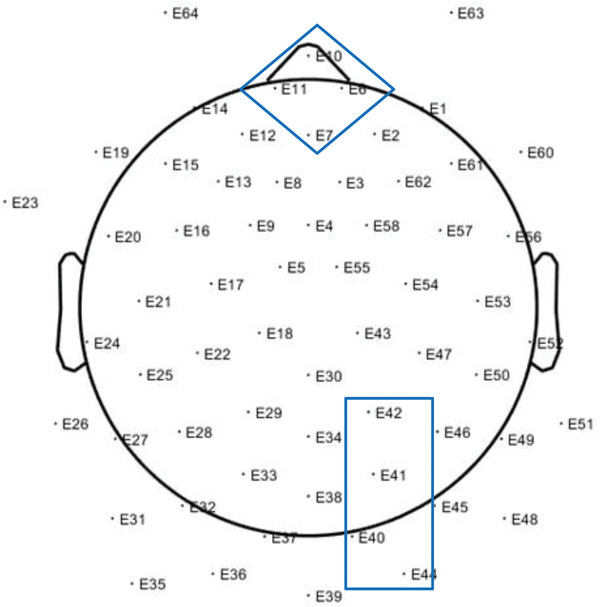
Channel groups used to analyse the frequency band between 3 and 9 Hz. The anterior channel group comprised of E6, E7, E10 and E11. The posterior channel group comprised of E40, E41, E42 and 44.

Power spectral density (henceforth power) is expressed as mean square microvolts and data were transformed using the natural log to normalise the distribution. Power was calculated for two electrode clusters selected to capture areas of interest identified in the literature review above: the anterior region (electrodes 6, 7, 10 and 11) and the posterior region (electrodes; 40, 41, 42 and 44; see Figure 1).

See SM1 for details of the method used for processing ERPs (analysis of which is also reported in SM1).

#### 2.2.3 A‐not‐B Task

Infants sat on their parent's lap centrally in front of a table containing two wells 11.2 cm in diameter and positioned 18 cm apart. An experimenter sat opposite to the infant, across the table, drew the infant's attention to a small colourful toy (from a selection available out of view of the infant) then placed the toy into one of the wells and covered both wells simultaneously. The side where the toy was hidden in the first trial was counterbalanced across participants. During a delay period (of initially 2 s), the experimenter drew the infant's attention away from the hiding location by talking in an exaggerated voice, saying the infant's name, waving and so forth, and the parent held the infant back to prevent them from reaching towards the wells. At the end of the delay period, the experimenter said ‘okay’, to signal to the parent to let the infant reach. If the infant reached to the correct well, they were allowed to briefly play with the toy. If the infant reached to the incorrect well, the experimenter drew the infant's attention to the toy in the correct well and started a new trial without letting them touch the toy. If the infant reached to both wells simultaneously or did not reach at all the trial was repeated. The toy was hidden in the same well until the infant reached to the correct location on two consecutive trials, at which point, the toy was hidden in the other well until the infant had completed another two successful trials consecutively. If two consecutive trials ended in failure, the delay period was decreased by 2 s. If two consecutive change trials ended in success, the delay period was increased by 1 s. The experiment was continued for 30 trials or until the infant stopped attending to the task.

##### 2.2.3.1 Derived Measure

As detailed in Holmboe et al. (2008), the performance metric for this task was calculated by summing the observed delays for all successful trials following a location change for each infant, and then dividing the result by the total number of trials completed by that infant.

### 2.3 Analytic Approach

To address our first research question ('Do we see different patterns in 3–6 or 6–9 Hz power according to looking behaviour during the Freeze‐Frame task?'), we conducted ANOVAs to test for effects of looking behaviour on EEG power in the Freeze‐Frame task. We conducted 2‐way ANOVAs including the factors of Condition (Looking vs. Not Looking to the distractor) and Location (Anterior vs. Posterior channel groups) and tested for a possible interaction between Condition and Location on absolute power. These ANOVAs were run separately for the 3–6 Hz and 6–9 Hz bands.

To address our second and third research questions (‘Do we find predictive associations from 3–6 or 6–9 Hz power within looking conditions on the Freeze‐Frame task at 6 months, to behavioural performance on the Freeze‐Frame/A‐not‐B task at 9 months’), where a significant condition effect was found, individual differences in within‐condition power were taken forward to longitudinal analyses. We tested for a predictive association with behavioural markers of attentional control and executive function at 9 months. Specifically, we used linear regression to test for an association between the neural marker at 6 months (the independent variable), and the behavioural marker (Freeze‐Frame performance, or A‐not‐B performance) at 9 months (the dependent variable), using separate linear regressions for each neural marker. Where a significant predictive association from the neural marker was found, step‐wise multiple linear regressions were run using both EEG and behavioural measures at 6 months to identify which predictors account for the most variance in 9‐month behaviour.

## 3 Results

### 3.1 Looking Condition Effects for EEG Power

**FIGURE 2:**
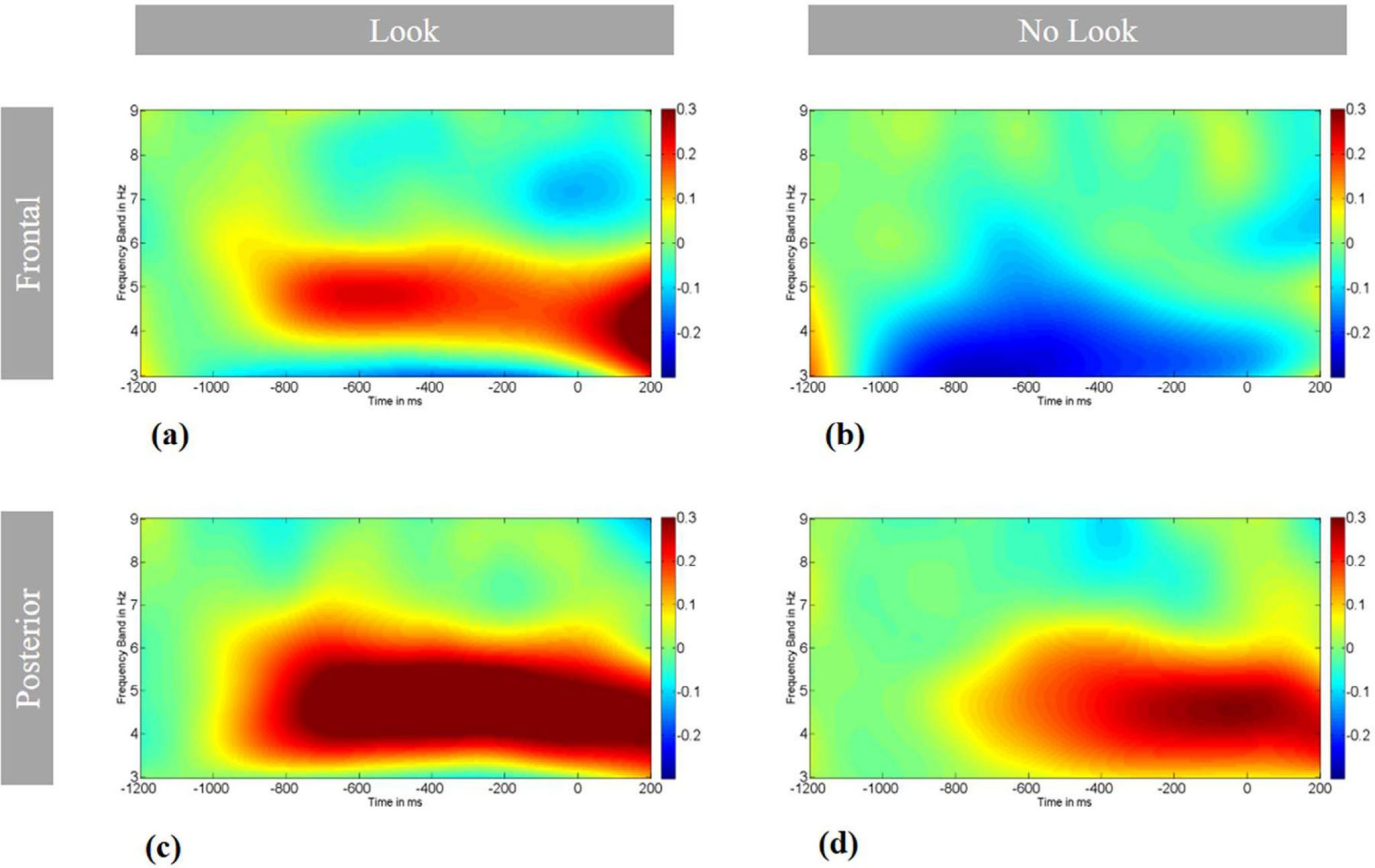
Power between 3 and 9 Hz during: (a) Look trials across anterior channels, (b) NoLook trials across anterior channels, (c) Look trials across posterior channels and (d) NoLook trials across posterior channels. The figures show the time from 1200 ms before distractor onset (i.e., time point 0) to 200 ms after distractor onset with saccades towards the distractor in the Look trials occurring after the 200 ms time point. The period from −1200 to −1000 ms was used as a baseline.

As shown in Table 3 and Figure 2, there was a medium effect of looking behaviour and a large effect of location on 3–6 Hz power, with no significant interaction between looking behaviour and location, such that 3–6 Hz power overall was greater in posterior channels (*M*Front = −0.04 [95% CI: −0.12, 0.03], *SD*Front = 0.5; *M*Post = 0.17 [95% CI: 0.07, 0.27], *SD*Post = 0.69; *M*Difference = 0.21 [95% CI: 0.11, 0.31]), and power during the Look trials was higher than during NoLook trials (*M*Look = 0.13 [95% CI: 0.04, 0.23], *SD*Look = 0.63; *M*NoLook = −0.01 [95% CI: −0.09, 0.07], *SD*NoLook = 0.56; *M*Difference = 0.14 [95% CI: 0.04, 0.25]. Post‐hoc paired samples *t*‐tests for 3–6 Hz power in anterior channels (specifically selected as our location of interest based on the literature summarised above indicating that 3–6 Hz power in anterior regions, in particular, is implicated in attentional control) confirmed that anterior 3–6 Hz power was greater during Look trials (*M* = 0.06, *SD* = 0.39) compared with the NoLook trials (*M* = −0.15, *SD* = 0.32) (*t*(1, 58) = 3.499, *p* = 0.001, Cohen's *d* = 0.59 [95% CI: 0.19, 0.72]). No significant main or interaction effects were found for the 6–9 Hz band.

**TABLE 3:** Analysis of variance in EEG power by looking behaviour and location.

3–6 Hz	*F*	Sig	Partial eta squared
Looking behaviour	7.336	0.009	0.112
Location	17.234	<0.001	0.229
Looking behaviour × location	1.783	0.187	0.030

6–9 Hz	*F*	Sig	Partial eta squared
Looking behaviour	1.287	0.261	0.022
Location	1.439	0.235	0.024
Looking behaviour × location	0.208	0.650	0.004

### 3.2 Neural Correlates of Attentional Control at 6 Months as a Predictor of Attentional Control and Executive Function at 9 Months

As shown in Table 4, 9‐month Freeze‐Frame behavioural performance (proportion of looks to distractors during interesting trials) was negatively associated with 3–6 Hz power (calculated relative to a baseline before the analysis period) during both Look and NoLook trials at 6 months—that is, infants who were less distractible at 9 months tended to have higher 3–6 Hz power when observing a dynamic stimulus at 6 months, regardless of whether they subsequently looked to a peripheral distractor. Exploratory follow‐up analysis shown in Table S2.1 indicates that the association between theta power during Look trials at 6 months and 9‐month Freeze‐Frame performance was specific to variation at 3 Hz. In Figure 2 in the 3 Hz band, we see some evidence for desynchronization (theta suppression) during Look trials—in contrast to the pattern of synchronisation observed in the 4–6 Hz range for Look trials.

**TABLE 4:** Prediction of 9‐month behavioural performance from 6‐month neural measures.

IV	DV	Beta	*β*	*F*	*df*	*p*	adj. *R2*
3–6 Hz during Look trials	9‐month FF	−0.091	−0.272	4.237	54	0.044	0.057
3–6 Hz during NoLook trials	9‐month FF	−0.115	−0.289	4.834	54	0.032	0.066
3–6 Hz during Look trials	9‐month A‐not‐B	0.355	0.117	0.689	51	0.410	−0.006
3–6 Hz during NoLook trials	9‐month A‐not‐B	0.097	0.026	0.035	51	0.853	−0.019

*Note*: FF = Freeze‐Frame performance (proportion of looks to distractors during interesting trials, a lower proportion indicates better performance).

Stepwise regression of 9‐month Freeze‐Frame behavioural performance on 6‐month 3–6 Hz power (Look and NoLook conditions entered separately), and 6‐month behavioural performance indicated that 3–6 Hz power during the NoLook condition was the best predictor of 9‐month behavioural performance, explaining 8% of the variance (*F*(1, 51) = 5.371, *p* = 0.025, Adj *R* ^2^ = 0.078); see also Table 5. For comparison, 6‐month behavioural performance alone explained 7% of the variance in 9‐month behavioural performance in the full sample (*F*(1, 70) = 6.650, *p* = 0.012, Adj *R* ^2^ = 0.074) and 4% in the sample with EEG data (*F*(1, 51) = 2.902, *p* = 0.095, Adj *R* ^2^ = 0.035). Follow‐up analysis showed that when 3 Hz power specifically was entered into the stepwise regression model for the Look condition, along with 3–6 Hz power in the NoLook condition and 6‐month behavioural performance, the model including power in both conditions but not 6‐month behavioural performance had the best prediction of 9‐month behavioural performance, explaining 16% of the variance (*F*(2, 50) = 6.080, *p* = 0.004, Adj *R* ^2^ = 0.163) (this is further illustrated in Table S2.2).

**TABLE 5:** Stepwise regression of 9‐month behavioural performance (Freeze‐Frame) on 6‐month neural and behavioural measures.

Model	IV	*t*	*p*	Beta	*Β*	*F*	*df*	*p*	adj. *R* ^*2*^	*R* ^*2*^ change
1	3–6 Hz during NoLook trials	−2.318	0.025	−0.309	−0.126	5.371	1.51	0.025	0.078	0.095
2	3–6 Hz during NoLook trials	−1.981	0.053	−0.263	−0.107	4.391	2.50	0.017	0.115	0.054
	3–6 Hz during Look trials	−1.784	0.081	−0.237	−0.080					
3	3–6 Hz during NoLook trials	−1.493	0.142	−0.206	−0.084	3.639	3.52	0.019	0.132	0.033
	3–6 Hz during Look trials	−1.957	0.056	−0.259	−0.087					
	6‐month FF performance	1.402	0.167	0.190	0.144					

*Note*: FF = Freeze‐Frame performance (proportion of looks to distractors during interesting trials), whereby a high FF performance score indicates less looking to the distractors. Tolerance > 0.87 and Variance inflation factors (VIF) < 1.15 for variables in all models.

As shown in Table 4, A‐not‐B performance was not significantly associated with 3–6 Hz power during either Look or NoLook trials. Similar results were found when a zero‐inflated Poisson model was used to account for the high proportion of infants with delays of 0 on the A‐not‐B task: The probability of not managing a delay greater than 0 s was not significantly associated with 3–6 Hz during Look trials (*p* = 0.761) or NoLook trials (*p* = 0.331), whilst for those who did manage some delay, delay duration was not significantly associated with 3–6 Hz during Look trials (*p* = 0.539) or NoLook trials (*p* = 0.239).

## 4 Discussion

### 4.1 Summary of Findings

We investigated the neural correlates of looking behaviour at 6 months of age during a task that elicits top‐down attentional control, as well as longitudinal associations between individual differences in those neural correlates at 6 months and attentional control and executive function performance at 9 months. In answer to our first research question (‘Do we see different patterns in 3–6 or 6–9 Hz power according to looking behaviour during the Freeze‐Frame task?’) we observed different patterns in 3–6 Hz—but not 6–9 Hz—power according to looking behaviour during the Freeze‐Frame task. Specifically, frontal 3–6 Hz synchronisation was greater in Look trials than in NoLook trials. In answer to our second and third research questions (‘Do we find predictive associations from 3–6 or 6–9 Hz power within looking conditions on the Freeze‐Frame task at 6 months, to behavioural performance on (i) the Freeze‐Frame task or (ii) the A‐not‐B task at 9 months’) infants who were less distractible at 9 months tended to have had, at 6 months of age, higher 3–6 Hz synchronisation in NoLook trials, and higher synchronisation in the 3 Hz band specifically in Look trials (this more‐precise band was identified through post‐hoc exploratory analysis). No significant longitudinal association was found to A‐not‐B performance. Below, we address our fourth question ‘How does the pattern of results identified above help us to interpret the attentional demands of the Freeze Frame task?’, and reflect on how our pattern of results fits within the extant literature on 3–6 and 6–9 Hz activation as neural indices of attentional control.

### 4.2 Interpretation

Our finding that looking behaviour during an attentional control task at 6 months varies with activity in the 3–6 Hz band, but not the 6–9 Hz band, is consistent with work by Marshall, Bar‐Haim, and Fox (2002) arguing that the 4–6 Hz band may be a better index of attentional control at 5 months, and work by Xie, Mallin, and Richards (2018) indicating that the classic ‘alpha’ desynchronization effect is evident within the 6–9 Hz band only from 10 months. Our findings also extend previous work linking frontal 3–6 Hz synchrony in infancy to the deployment of endogenous attention and information processing (Begus, Southgate, and Gliga 2015; Braithwaite et al. 2020; Brandes‐Aitken et al. 2023; Jones et al. 2020; Köster, Langeloh, and Hoehl 2019; Köster et al. 2021; Michel et al. 2015; Orekhova et al. 2006; Piccardi, Johnson, and Gliga 2020; Stroganova and Orekhova 2013; Wass et al. 2018; Xie, Mallin, and Richards 2018) by demonstrating that 3–6 Hz frontal power in infants is implicated during a task that is considered to engage attentional control. As shown in Figure 2, and as would be expected based on the literature, condition effects were most obvious in frontal rather than posterior channels. However, the interaction between location and condition was not significant, indicating either that posterior 3–6 Hz activity is also implicated in attentional control, or insufficient power to detect an interaction. To limit the number of multiple comparisons, and in line with the literature, we selected only frontal 3–6 Hz power for the longitudinal analyses discussed below.

One key finding was that frontal 3–6 Hz synchronisation was greater in Look trials than in NoLook trials. To interpret this finding, it is important to consider that, in the version of the task used during EEG recording, the central stimulus briefly freezes on presentation of the distractor for all trials and there is thus no cost to looking to the peripheral stimulus.

Moreover, the peripheral stimulus, although jittered to minimise time‐locked anticipatory processes, and randomised by side, appeared on every trial. Thus, although infants could not plan a saccade to a specific location at a specific time, our group‐level pattern of frontal 3–6 Hz synchronisation in Look trials could be indicative of general anticipation of the peripheral stimulus or at the very least of engagement with the task and readiness to orient to new stimuli as they appear. This interpretation is consistent with previous research indicating that theta power increases are a marker of readiness to process information in infancy (Köster, Langeloh, and Hoehl 2019; Köster et al. 2021; Wass et al. 2018): Anticipatory effects have previously been found for infants to be specifically in the 4–5 Hz range (Köster, Langeloh, and Hoehl 2019; Köster et al. 2021), and it is in this frequency range where we see the strongest evidence for power increases in Look trials (see Figure 2).

Meanwhile, in our data, at 3 Hz there was some evidence of desynchronisation, more akin to the pattern observed in the NoLook trials (see Figure 2). Potentially then, two different cognitive processes are indexed by EEG across the 3–6 Hz range: synchronization in the 4–6 Hz range may relate to anticipatory attention or engagement and be particularly evident in the trials where infants subsequently looked to the distractor; whilst the 3–4 Hz range may be sensitive to some other process. The pattern of relations between EEG at 6 months and later performance on the Freeze‐Frame task could be key to interpreting this latter process. In the 9‐month version of the task, where there is a cost to looking to the peripheral distractor (the more‐complex, dynamic central stimulus freezes for a longer period if the infant looks to the distractor), greater attentional control is indexed by fewer looks to the distractor. We found that infants who were less distractible at 9 months tended to have had, at 6 months of age, higher 3–6 Hz synchronisation in NoLook trials, and higher synchronisation in the 3 Hz band specifically in Look trials (this more‐precise band was identified through post‐hoc exploratory analysis). Why a slightly different pattern for Look versus NoLook trials? If we accept our earlier interpretation that anticipatory processes were implicated in Look trials (i.e., trials where infants subsequently look to a peripheral distractor), it could be that for those trials anticipatory processes ‘drown out’ other aspects of attentional control (such as those involved in staying on task) in the 4–6 Hz range (but not at 3 Hz). It is important to note that these are exploratory findings, and therefore must be considered only preliminary; nevertheless, this is the first indication that brain activity during an attentional control task at 6 months is predictive of looking behaviour in later infancy and worthy of further investigation.

Importantly, we find that the predictive association between frontal 3–6 Hz power during NoLook trials at 6 months shows a stronger predictive relation to looking behaviour at 9 months than looking behaviour at 6 months alone: 3–6 Hz power explains 8% of variance in 9‐month Freeze‐Frame performance, whereas looking behaviour alone explains 4% of the variance within the same sub‐sample. When 3 Hz activity during Look trials was also included in the regression, infant EEG at 6 months explained 16% of variance in 9‐month Freeze‐Frame performance. These findings provide support for previous suggestions that frontal 3–6 Hz activity may be useful as a biomarker of attentional control or information encoding (Jones et al. 2020). They also indicate that predictive associations from frontal 3–6 Hz activity to later indices of attentional control can be found from as early as 6 months of age. This predictive association contrasts with the lack of predictive association to attentional control at 9 months observed for 3–6 Hz power recorded from 6‐month‐olds during the presentation of a non‐social video with no particular demands on attentional control (Braithwaite et al. 2020), indicating that the context in which infant EEG is recorded is key.

We hesitate however to go so far as to say that 3–6 Hz activity at 6 months could be a precise marker of executive attention, as we found no predictive association from 3–6 Hz activity to 9‐month A‐not‐B performance. Of course, it is possible that the influence of processes and mechanisms other than executive attention on A‐not‐B performance (see Hendry et al. 2021, for discussion) add sufficient noise to the performance measure to mask potential predictive associations from the infant EEG to A‐not‐B performance. Indeed, as mentioned in the Introduction, previous work has found only weak associations between Freeze‐Frame performance at 6 months and A‐not‐B performance at 9 months, which did not meet standard significant thresholds even in the full cohort (Holmboe et al. 2018). However, concurrent associations between 9‐month A‐not‐B performance and Freeze‐Frame performance in the same sample have previously been reported, which are presumed to be attributable to individual differences in executive processes (specifically inhibitory control) (Holmboe et al. 2018). Instead, we suggest that the differential association between 6‐month EEG and 9‐month Freeze‐Frame versus A‐not‐B performance should be interpreted through the lens of the different task demands: In the 9‐month version of the task, which freezes when infants orient to the peripheral stimulus, infants are encouraged via the task design to inhibit looks to these peripheral distractor; therein lie the primary executive demands of the task, along with the attentional demands of the task inherent in needing to maintain attention on the screen. In the 6‐month version of the task, there are still the attentional control demands required to maintain attention on the screen (and, we suggest on the basis of the observed condition‐effects, some anticipatory processes being engaged), but there is a lower initial cost to looking to the peripheral stimulus. Over time it does appear to be the case that (as intended) infants find the repetitive, simple peripheral stimuli less engaging and, thus, learn to inhibit looks to these distractors—however, the inhibitory demands of this learned response may be lower than in the 9‐month version. Thus, it seems likely the case that the associations observed between 6‐month EEG and looking behaviour during the Freeze‐Frame task, and 9‐month looking behaviour during the Freeze‐Frame task are attributable to continuity in attentional control, whereas the association previously reported between 9‐month A‐not‐B performance and concurrent Freeze‐Frame looking behaviour is attributable to individual differences in additional executive processes. Our analyses provide no evidence that these executive processes are associated with 6‐month EEG power and, as reported in SM1, group‐level analyses found no significant condition effects on the P1–our ERP marker of inhibitory gating at 6 months (Klimesch 2011; Slagter et al. 2016). This means we have no evidence to support the argument that infants engage in active inhibitory processes in response to the distractor to maintain attention on the central stimulus at 6 months. However, general attentional control (i.e., focused attention) is still needed to maintain attention to the screen; variation in which is reflected in the pre‐distractor 3–6 Hz activity, and longitudinal associations.

### 4.3 Implications and Limitations

Lowering the age at which atypicalities in cognitive development can be detected has important implications for translational research, where early intervention may potentially yield the greatest impacts (Wass, Scerif, and Johnson 2012). Nevertheless, the predictive association of 3–6 Hz power to later attentional control in our data was still modest, and considerable further work is required to refine both the statistical model and the experimental design before it approaches the sensitivity needed to be a useful biomarker. Indeed, although it seems likely from this and previous reports that EEG is in some ways a purer measure of attentional control than infant behaviour (which is notoriously noisy and likely to be influenced by many factors and processes beyond just attentional control) (Hendry, Johnson, and Holmboe 2019), the fact that not all infants will contribute useable EEG data remains a limitation for this line of research (Begus and Bonawitz 2020). In this study, our attrition rate of 39% compares favourably to the average attrition rate for ERPs studies (49%) (Stets, Stahl, and Reid 2012), but is not as low as attrition rates achieved for EEG in a more‐recent large infant neuroimaging study (27%) (van der Velde and Junge 2020). Due to the common self‐regulatory requirements involved in complying with a novel, mildly aversive situation (wearing an EEG cap), sitting still long enough to provide sufficient valid trials on a repetitive screen‐based (Freeze‐Frame) or behavioural (A‐not‐B) task, and inhibiting looks to a peripheral distractor (i.e., performance on the Freeze‐Frame task), it is likely that 9‐month data are not missing at random. Rather than break the assumptions of maximum likelihood estimation (which would allow us to leverage the full initial sample but requires data to be missing at random), we ran each longitudinal model using only those infants with available study data. As a consequence, a key limitation of this study is the modest sample contributing longitudinal data which may not be representative of the general infant population (but rather be representative of infants who have the regulatory capacity to tolerate EEG net placement and maintain engagement with a repetitive task). Moreover, our modest sample limits our statistical power; we achieved 54%–60% power to detect the effect sizes observed for predictive associations between 6‐month neural measures and 9‐month Freeze‐Frame performance, but had less than 20% power to detect small predictive associations to 9‐month A‐not‐B performance.

## 5 Conclusions

The data presented in this study corroborate and extend prior research linking 3–6 Hz frontal power to attentional control in infancy. By considering differences in EEG in different conditions on a task designed to elicit attentional control at 6 months, as well as longitudinal associations from 6‐month EEG to behavioural performance at 9 months, we have identified that this band may be sensitive to multiple processes, such as anticipatory attention, and the ability to maintain attention on a target. Further, we demonstrate that predictive associations from frontal 3–6 Hz activity to later indices of attentional control can be found from as early as 6 months of age.

## Conflicts of Interest

The authors declare no conflicts of interest.

## Supporting information



Supporting‐Information

## Data Availability

The data underlying the analyses reported in this article are available on OSF: https://osf.io/cnr3u/.
