# Supplementary material for: Neural Markers of Attention at 6 Months Associate With Later Attentional Control Performance
Source: Dev Sci. 2024 Nov 6;28(1):e13582. doi: 10.1111/desc.13582 (PMC12403191; doi:10.1111/desc.13582)
Supplement: Supplementary file 1 — Supporting‐Information [file DESC-28-e13582-s001.docx]

# Supplementary Materials 1: Neural markers of attention at 6 months associate with later attentional control performance; the P1 Event-Related Potential

# Rationale

An alternative or complement to considering EEG power as a neural index of attentional control is to identify the time-locked EEG waveform components – known as Event-Related Potentials (ERPs) – associated with attentional control. Several ERP components have been implicated in top-down modulation of visual attention, but as the experimental attention task used in this study involves stimuli presented at the periphery which, therefore, elicit eye movement artefacts in the EEG, we focus here on waveform components occurring rapidly after distractor onset but before a voluntary saccade can be performed; i.e. the P1.

The P1 has been described as the earliest ERP index of endogenous visual attention, reflecting the interaction of bottom‐up sensory processing (via frontal‐posterior attention networks) with top-down attentional control (Hillyard, Vogel, & Luck, 1998; Luck, Heinze, Mangun, & Hillyard, 1990; Taylor, 2002). Specifically, P1 is considered to reflect selection influenced by the context of the sensory, historical, or goal saliency of a stimulus via top‐down suppression (Finnigan, O'Connell, Cummins, Broughton, & Robertson, 2011; Jones, Dawson, & Webb, 2018; Natale, Marzi, Girelli, Pavone, & Pollmann, 2006; Zanto & Gazzaley, 2009) and is sensitive to task demands in both spatial and non-spatial visual tasks, amongst children and adults. For example, P1 amplitude is larger in conditions of active versus passive viewing (Fu, Fedota, Greenwood, & Parasuraman, 2010), and is affected by task load (Taylor, 2002) as well as by participants’ attention to the stimulus (Mangun, 1995; Taylor & Khan, 2000). The P1 typically occurs over lateral occipital sites and peaks around 100ms after stimulus onset in adults. Latencies increase with processing demands and decrease with age such that P1 peaks can be expected to occur later for infants in comparison to adults (Taylor, 2002). As well as indexing general attentional modulation, the P1 may be particularly sensitive to inhibitory gating (Klimesch, 2011; Slagter, Prinssen, Reteig, & Mazaheri, 2016). In adults, distractor-induced P1 amplitudes are reduced when those distractors can be predicted (Noonan et al., 2016). Modulations of the P1 during inhibitory control tasks have been associated with functional neuroanatomical structures in the response inhibition network (Chmielewski & Beste, 2019; Giller, Zhang, Roessner, & Beste, 2019; Wolff, Giller, Buse, Roessner, & Beste, 2018).

Amongst infants, the P1 has been consistently linked to modulation of attention using spatial cueing paradigms (Richards, 2000, 2005). Most recently, Xie and Richards (2017) have found that amongst 3- to 4.5-month-olds, spatial-cueing effects on the P1 response are greatest during periods of heart-rate defined sustained attention. To our knowledge, there are no studies considering the role of P1 during contrasting conditions of looking behaviour.

# Method

Participants and Freeze-Frame procedure are as per the main manuscript. Of the original 96 infants, 62 (34 female) provided sufficient artefact-free EEG recordings at 6 months to compute ERPs.

## ERP processing

Using the EEGLab software package, ERPs were sampled between 200 ms before and 800 ms after distractor-onset using the period between -200 ms and 0 ms as the baseline. Epochs were visually inspected and rejected if trials had been marked as invalid during video-coding or if the data were too noisy due to movement or other artefacts. An infant’s data were included in the final analysis if they reached a minimum criterion of having 8 valid trials for both the Look and the NoLook condition.

Infants included in the final analysis contributed an average of 18.31 artefact-free Look trials (*SD*=7.87, range 8 to 40, total 1,135) and an average of 34.11 artefact-free NoLook trials (*SD*=18.52, range 8 to 89, total 2,115). The discrepancy between usable trials for the Look and the NoLook conditions is due to infants looking to distractors less often than not, as well as more Look trials having to be excluded from the analysis due to eye-movement artefacts.

Based on the individual and grand-averaged data, as well as previous literature (Richards, 2000; Xie & Richards, 2017), the peak amplitude of the P1 was extracted within a time window of 80-180 ms after distractor onset. The laterality of the P1-peak is connected to the side on which a distractor has been presented, e.g., for distractors presented on the left side of the screen, the P1 is expected to be more pronounced over the right hemisphere and vice versa (Mangun, Hillyard, & Luck, 1993). Therefore, to facilitate data analysis, for trials in which the distractor had been presented on the right side, channels were mirrored along the vertical axis such that for the purpose of data-analysis all distractors were presented on the left. In the following, data were coded as ipsilateral or contralateral; i.e., with the distractor presented on the left, left-hemisphere channels were coded as ipsilateral, and right hemisphere channels as contralateral. The channels selected to form the ipsilateral group were E22, E28, E29, and E32. The channels selected to form the contralateral group were E42, E45, E46, and E47, respectively (see Supplementary Figure 1).


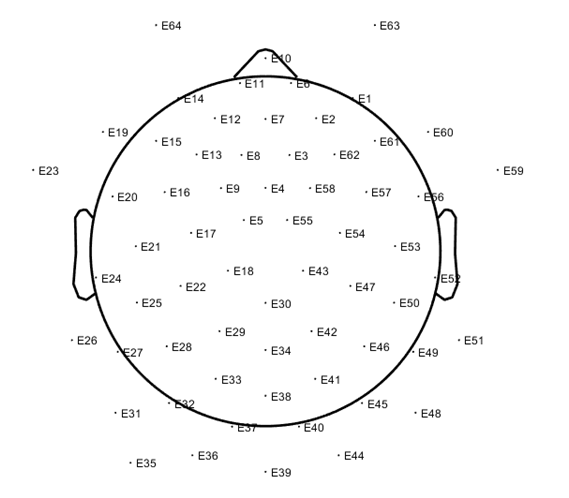


**Supplementary Figure 1.** Channel groups used in the analysis of the P1-component. The ipsilateral channel group comprised of E22, E28, E29, and E32. The contralateral channel group comprised of E42, E45, E46, and E47.

## Analytic approach

As per the approach for EEG analysis, we first conducted ANOVAs to test for effects of looking behaviour on P1 amplitude in the Freeze-Frame task. We conducted a 2-way ANOVA testing for a possible effect of Condition (Looking vs Not Looking to the distractor) and Location (ipsilateral or contralateral) as well as for an interaction between Condition and Location.

# Results

### P1 condition effects

As shown in Supplementary Table 1 and Supplementary Figure 2, there were no significant effects of looking behaviour (Look or NoLook) or laterality (ipsilateral or contralateral) on P1 amplitude, and no significant interaction between looking behaviour and location. Average amplitudes for the P1 between 80 and 180ms after distractor-onset for Look trials were as follows: *M_Ipsi_* = 0.95, *SD_Ipsi_* = 5.27 and *M_Contra_* = 1.6, *SD_Contra_* = 4.25. Average amplitudes for the P1 between 80 and 180ms after distractor-onset for NoLook trials were as follows: *M_Ipsi_* = 0.29, *SD_Ipsi_* = 2.82 and *M_Contra_* = 0.82, *SD_Contra_* = 2.73.

Supplementary Table 1. Analysis of Variance in P1 amplitude by looking behaviour and condition

|  | F | Sig | Partial eta squared |
| --- | --- | --- | --- |
| Looking Behaviour | 1.659 | .203 | 0.026 |
| Laterality | 2.235 | .140 | 0.035 |
| Looking Behaviour * Laterality | 0.021 | .886 | 0.000 |

|  |  |
| --- | --- |
| 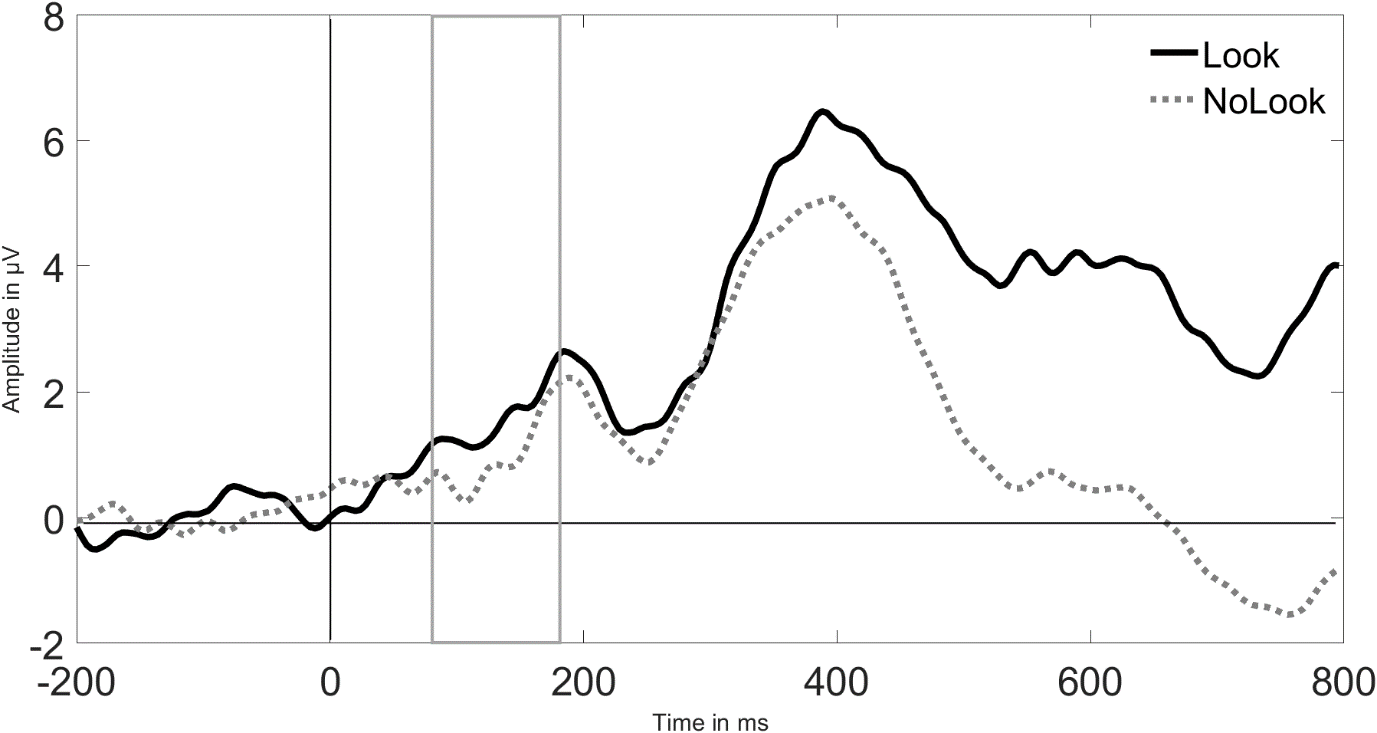  (a) |  |


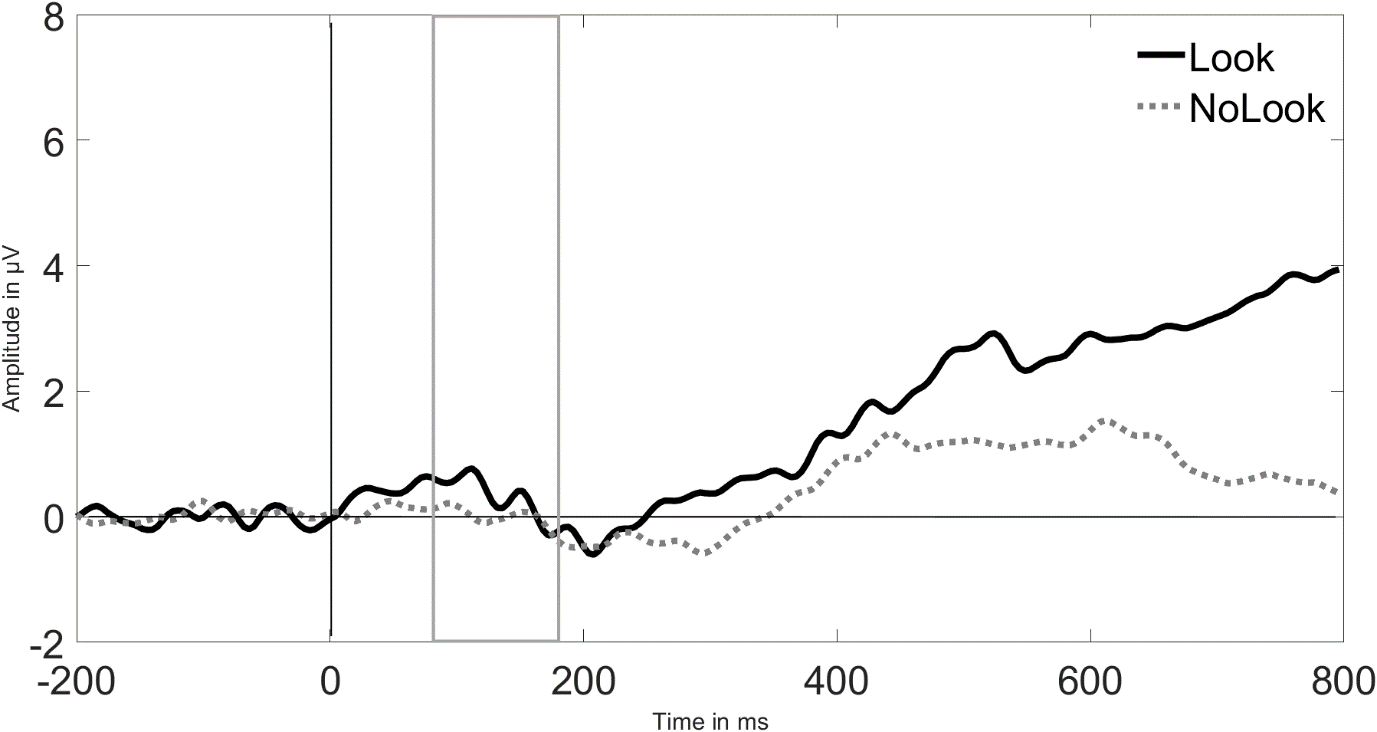


(b)

Supplementary Figure 2. ERP waveform in (a) contralateral (E42, E45, E46, and E47) and (b) ipsilateral (E22, E28, E29, and E32) channels for 62 infants. The depicted time course covers the 200 ms baseline period before distractor onset (i.e., timepoint 0) up to 800 ms after distractor onset. The time window selected for statistical analysis stretched from 80 to 180 ms after distractor onset and as indicated by the grey box in Supplementary Figure 2.

# Supplementary Materials 2: Additional EEG power analyses

Supplementary Table 2.1. Linear regression of 9-month Freeze-Frame performance on 6-month neural measures, by 1Hz frequency bands (n=55)

| IV | Beta | *β* | *F* | *p* | adj. *R^2^* |
| --- | --- | --- | --- | --- | --- |
| 3Hz during Look trials | -.079 | -.310 | 5.634 | .021 | .079 |
| 4Hz during Look trials | -.029 | -.132 | 0.935 | .338 | -.001 |
| 5Hz during Look trials | -.041 | -.204 | 2.297 | .136 | .023 |
| 6Hz during Look trials | -.055 | -.150 | 1.221 | .274 | .004 |
|  |  |  |  |  |  |
| 3Hz during NoLook trials | -.028 | -.123 | 0.816 | .370 | -.003 |
| 4Hz during NoLook trials | -.053 | -.238 | 3.187 | .080 | .039 |
| 5Hz during NoLook trials | -.061 | -.215 | 2.569 | .115 | .028 |
| 6Hz during NoLook trials | -.111 | -.244 | 3.349 | .073 | .042 |

Supplementary Table 2.2. Multiple regression (Step-wise method) of 9-month behavioural Freeze-Frame performance on 6-month neural (3Hz during look trials specifically) and behavioural measures

| Model | IV | *T* | *p* | Beta | *Β* | *F* | *df* | *p* | adj. *R^2^* | *R^2^* change |
| --- | --- | --- | --- | --- | --- | --- | --- | --- | --- | --- |
| 1 | 3-6Hz during NoLook trials | -2.318 | .025 | -.309 | -.126 | 5.371 | 1,51 | .025 | .078 | .095 |
| 2 | 3-6Hz during NoLook trials | -2.236 | .030 | -.284 | -.116 | 6.080 | 2,50 | .004 | .163 | .100 |
|  | 3Hz during Look trials | -2.497 | .016 | -.318 | -.086 |  |  |  |  |  |
| 3 | 3-6Hz during NoLook trials | -1.813 | .076 | -.239 | -.097 | 4.601 | 3,52 | .006 | .172 | .024 |
|  | 3Hz during Look trials | -2.525 | .015 | -.320 | -.086 |  |  |  |  |  |
|  | 6-month FF performance | 1.232 | .224 | .162 | .123 |  |  |  |  |  |

# Supplementary Materials References

Chmielewski, W. X., & Beste, C. (2019). Stimulus-response recoding during inhibitory control is associated with superior frontal and parahippocampal processes. *Neuroimage, 196*, 227-236. doi:<https://doi.org/10.1016/j.neuroimage.2019.04.035>

Finnigan, S., O'Connell, R. G., Cummins, T. D., Broughton, M., & Robertson, I. H. (2011). ERP measures indicate both attention and working memory encoding decrements in aging. *Psychophysiology, 48*(5), 601-611.

Fu, S., Fedota, J. R., Greenwood, P. M., & Parasuraman, R. (2010). Dissociation of visual C1 and P1 components as a function of attentional load: An event-related potential study. *Biological Psychology, 85*(1), 171-178. doi:<https://doi.org/10.1016/j.biopsycho.2010.06.008>

Giller, F., Zhang, R., Roessner, V., & Beste, C. (2019). The neurophysiological basis of developmental changes during sequential cognitive flexibility between adolescents and adults. *Human Brain Mapping, 40*(2), 552-565. doi:10.1002/hbm.24394

Hillyard, S. A., Vogel, E. K., & Luck, S. J. (1998). Sensory gain control (amplification) as a mechanism of selective attention: electrophysiological and neuroimaging evidence. *Philosophical Transactions of the Royal Society of London. Series B: Biological Sciences, 353*(1373), 1257-1270.

Jones, E. J. H., Dawson, G., & Webb, S. J. (2018). Sensory hypersensitivity predicts enhanced attention capture by faces in the early development of ASD. *Developmental Cognitive Neuroscience, 29*, 11-20. doi:<https://doi.org/10.1016/j.dcn.2017.04.001>

Klimesch, W. (2011). Evoked alpha and early access to the knowledge system: The P1 inhibition timing hypothesis. *Brain Research, 1408*, 52-71. doi:<https://doi.org/10.1016/j.brainres.2011.06.003>

Luck, S. J., Heinze, H. J., Mangun, G. R., & Hillyard, S. A. (1990). Visual event-related potentials index focused attention within bilateral stimulus arrays. II. Functional dissociation of P1 and N1 components. *Electroencephalography and Clinical Neurophysiology, 75*(6), 528-542. doi:<https://doi.org/10.1016/0013-4694(90)90139-B>

Mangun, G. R. (1995). Neural mechanisms of visual selective attention. *Psychophysiology, 32*(1), 4-18.

Mangun, G. R., Hillyard, S. A., & Luck, S. J. (1993). Electrocortical substrates of visual selective attention.

Natale, E., Marzi, C. A., Girelli, M., Pavone, E. F., & Pollmann, S. (2006). ERP and fMRI correlates of endogenous and exogenous focusing of visual-spatial attention. *European Journal of Neuroscience, 23*(9), 2511-2521. doi:10.1111/j.1460-9568.2006.04756.x

Noonan, M. P., Adamian, N., Pike, A., Printzlau, F., Crittenden, B. M., & Stokes, M. G. (2016). Distinct mechanisms for distractor suppression and target facilitation. *Journal of Neuroscience, 36*(6), 1797-1807.

Richards, J. E. (2000). Localizing the development of covert attention in infants with scalp event-related potentials. *Developmental Psychology, 36*(1), 91.

Richards, J. E. (2005). Localizing cortical sources of event‐related potentials in infants’ covert orienting. *Developmental Science, 8*(3), 255-278.

Slagter, H. A., Prinssen, S., Reteig, L. C., & Mazaheri, A. (2016). Facilitation and inhibition in attention: Functional dissociation of pre-stimulus alpha activity, P1, and N1 components. *Neuroimage, 125*, 25-35. doi:<https://doi.org/10.1016/j.neuroimage.2015.09.058>

Taylor, M. J. (2002). Non-spatial attentional effects on P1. *Clinical Neurophysiology, 113*(12), 1903-1908. doi:<https://doi.org/10.1016/S1388-2457(02)00309-7>

Taylor, M. J., & Khan, S. C. (2000). Top-down modulation of early selective attention processes in children. *International Journal of Psychophysiology, 37*(2), 135-147. doi:<https://doi.org/10.1016/S0167-8760(00)00084-2>

Wolff, N., Giller, F., Buse, J., Roessner, V., & Beste, C. (2018). When repetitive mental sets increase cognitive flexibility in adolescent obsessive–compulsive disorder. *Journal of Child Psychology and Psychiatry, 59*(9), 1024-1032. doi:10.1111/jcpp.12901

Xie, W. Z., & Richards, J. E. (2017). The relation between infant covert orienting, sustained attention and brain activity. *Brain topography, 30*(2), 198-219.

Zanto, T. P., & Gazzaley, A. (2009). Neural Suppression of Irrelevant Information Underlies Optimal Working Memory Performance. *The Journal of Neuroscience, 29*(10), 3059-3066. doi:10.1523/jneurosci.4621-08.2009
